# Biomarkers of hepatocellular synthesis in patients with decompensated cirrhosis

**DOI:** 10.1007/s12072-022-10473-x

**Published:** 2023-01-18

**Authors:** Berivan Gurbuz, Nurdan Guldiken, Philipp Reuken, Lei Fu, Katharina Remih, Christian Preisinger, Radan Brůha, Martin Leníček, Jaromír Petrtýl, Johanna Reissing, Mahmoud Aly, Malin Fromme, Biaohuan Zhou, Isabel Karkossa, Kristin Schubert, Martin von Bergen, Andreas Stallmach, Tony Bruns, Pavel Strnad

**Affiliations:** 1 Department of Internal Medicine III and IZKF, Gastroenterology, Metabolic Diseases and Intensive Care, University Hospital Aachen, Pauwelsstraße 30, 52074 Aachen, Germany; 2 Department of Internal Medicine IV, Jena University Hospital, Friedrich Schiller University, Jena, Germany; 3 Department of Science and Technology, Ruikang Hospital Affiliated to Guangxi University of Chinese Medicine, Guangxi Zhuang Autonomous Region, Nanning, 530011 China; 4 Proteomics Facility, Interdisciplinary Center for Clinical Research (IZKF), University Hospital RWTH, Aachen, Germany; 5 4th Department of Internal Medicine, First Faculty of Medicine, General University Hospital in Prague, Charles University, Prague, Czech Republic; 6 Institute of Medical Biochemistry and Laboratory Diagnostics, First Faculty of Medicine, General University Hospital in Prague, Charles University, Prague, Czech Republic; 7 Department of Medicine and Infectious Diseases, Faculty of Veterinary Medicine, University of Sadat, 12 City, Sadat City, Egypt; 8 Department of Surgical Oncology, Fujian Provincial Hospital, Fuzhou, China; 9 Department of Molecular Systems Biology, Helmholtz Centre for Environmental Research, Leipzig, Germany; 10 German Centre for Integrative Biodiversity Research, (iDiv) Halle-Jena-Leipzig, Leipzig, Germany; 11 Faculty of Life Sciences, Institute of Biochemistry, University of Leipzig, Leipzig, Germany

**Keywords:** Mass spectrometry, Fibrosis, Interleukin, Hepatocyte nuclear factor, Decompensated cirrhosis

## Abstract

**Background and aim:**

Since hepatocytes produce majority of serum proteins, patients with cirrhosis display substantial alterations in the serum proteome. The aim of the current study was to characterize these changes and to study the prognostic utility of hepatocellular proteins available in routine clinical testing.

**Methods:**

Sera from 29 healthy controls and 43 patients with cirrhosis were subjected to untargeted proteomic analysis. Unsupervised hierarchical clustering was performed with Perseus software and *R*. Ingenuity pathway analysis (IPA) suggested upstream regulators that were validated in liver tissues. The behavior and prognostic usefulness of selected biomarkers was investigated in 61 controls and 285 subjects with decompensated cirrhosis.

**Results:**

Proteomics uncovered 65 and 16 hepatocellular serum proteins that are significantly downregulated or upregulated in patients with cirrhosis vs. controls. Hierarchical clustering revealed two main clusters and six sub-clusters. IPA identified HNF4α and IL-6 as the two major upstream regulators that were confirmed by hepatic gene expression analyses. Among pseudocholinesterase, transferrin, transthyretin, albumin, and apolipoprotein AI (Apo-AI), Apo-AI was the best predictor of 90-days transplant-free survival (AUROC 0.678; *p* = 0.0001) and remained an independent predictor in multivariable Cox independently of the presence of acute-on-chronic liver failure.

**Conclusion:**

Our study reveals cirrhosis-associated changes in hepatocellular serum proteins and underlying transcription factors. Serum apolipoprotein AI may constitute a useful prognostic adjunct in patients with decompensated cirrhosis.

**Graphical abstract:**

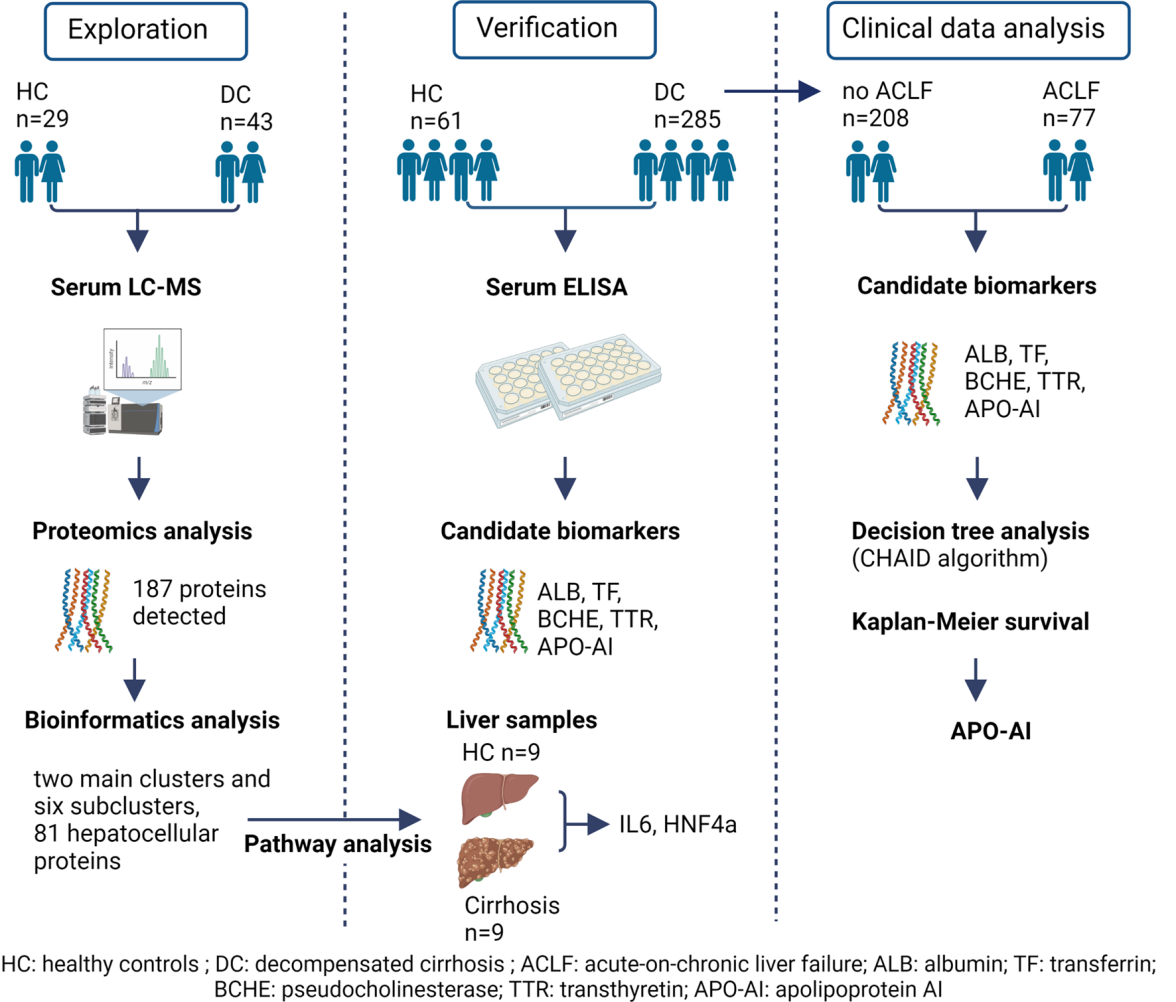

**Supplementary Information:**

The online version contains supplementary material available at 10.1007/s12072-022-10473-x.

## Introduction

Liver constitutes a central metabolic hub of the human body [1]. It receives nutrients from the intestine as well as compounds from peripheral tissues that are either stored or used for synthesis of new products [1]. Hepatocytes, the parenchymal cells of the liver, are responsible for these processes. They constitute highly active secretory cells and produce majority of proteins found in the serum including hormones, carrier and homeostatic proteins [2]. Protein synthesis is directed by a concerted action of liver-enriched transcription factors [3]. This teamwork enables the generation of multiple carrier and homeostatic proteins that are essential for organismal wellbeing. At the same time, it is responsible for adjustment to stress situations and because of that it responds to inflammatory stimuli [3, 4] It leads to a decreased generation of compounds that are deemed dispensable (so called anti-acute-phase proteins) and increased manufacturing of mediators supposed to help with the stress (termed acute-phase proteins, APPs) [2, 5] APPs are further subdivided into type I and type II, depending on whether they are regulated by interleukin 1-like cytokines such as interleukin-1α/β and tumor necrosis factor or IL-6 like cytokines such as IL-6, IL-11, oncostatin M and leukemia inhibitory factor.

Cirrhosis is the result of an evolutionarily conserved wound-healing response of the liver to tissue injury, usually triggered by inflammatory or immune-mediated mechanisms resulting in a loss of hepatocytes and the remodeling of the tissue architecture [6]. Capillarization of the sinusoids promotes hypoxia, which further impairs the synthetic ability of hepatocytes perpetuating injury and the release of damage-associated molecular patterns (DAMPs) [7]. In parallel, portal hypertension, intestinal dysbiosis, and impaired gut barrier function promote translocation of pathogen-associated molecular patterns (PAMPs) into the circulation [8].

In summary, cirrhosis is characterized by a hepatocellular loss together with hepatocellular re-programming triggered by hepatocellular de-differentiation, hypoxia, and innate immune activation [7, 8]. Since production of secreted proteins constitutes one of the key functions of the liver and changes in serum proteome can be easily measured, serum levels of hepatocellular proteins are potentially useful biomarkers reflecting the functional state of the liver. Notably, some of them, such as albumin or coagulation factors, became well-established components of prognostic scores [9]. Despite that, the exact alterations in hepatocellular serum proteome occurring in advanced liver disease still remain to be systematically characterized. Therefore, we performed an unbiased proteomic analysis of sera from cirrhotic patients as well as corresponding controls and used a bioinformatics approach to elucidate the biological pathways responsible for the observed changes. To validate the bioinformatics predictions, the results were corroborated via RNA expression analyses and the prognostic ability of selected hepatocellular products was studied in an independent cohort of patients with decompensated cirrhosis.

## Experimental procedures

### Patient cohorts

In total, frozen serum samples from 285 patients with acute decompensation (AD) of cirrhosis and from 61 self-identified healthy individuals were used for this study. Of these, sera from 21 patients with acute chronic liver failure (ACLF) or pre-ACLF, 22 patients with stable decompensated cirrhosis (SDC) according to the PREDICT study [10], and 29 healthy individuals were randomly selected for proteomic analysis (Table 1). Patient samples were derived from patients hospitalized for acute decompensation of cirrhosis treated at the Jena University Hospital between 09/2010 and 07/2015 as described previously [11] and frozen at − 80 °C until analysis. Sera from self-declared healthy individuals were collected at the University Hospital Aachen, Germany between the years 2016 and 2019 as described previously [12].

**Table 1:** Baseline characteristics

	Proteome analysis		Candidate biomarker analysis	
	Decompensated cirrhosis (*n* = 43)	Healthy subjects (*n* = 29)	Decompensated cirrhosis (*n* = 285)	Healthy subjects (*n* = 61)
Age (yrs)	53 (47–62)	57 (53–61)	59 (53–68)	61 (56–67)
Male sex (%)	38 (88)	21 (72)	210 (74)	45 (74)
Alcohol-related liver disease (%)	39 (91)	0	227 (80)	0
Ascites	43 (100)	0	285 (100)	0
ACLF	13 (30)	0	77 (27)	0
Pre-ACLF	9 (21)	0	66 (23)	0
Hepatocellular carcinoma	7 (16)	0	40 (14)	0
MELD score	16 (12–25)	N/A	17 (12–22)	N/A
Bilirubin (µmol/L)	58 (23–184)	10 (6–13)	46 (24–109)	8 (6–13)
ALT (µmol/[L × s])	0.5 (0.4–1.3)	0.5 (0.4–0.6)	0.6 (0.4–0.9)	0.4 (0.3–0.5)
AST (µmol/[L × s])	1.0 (0.4–1.3)	0.4 (0.4–0.5)	1.1 (0.7–1.9)	0.4 (0.4–0.5)
INR	1.5 (1.3–1.9)	0.9 (0.9–1)	1.4 (1.2–1.7)	0.9 (0.9–1)
Platelets (/nL)	137 (96–178)	244 (206–285)	124 (80–177)	224 (187–284)
Creatinine (µmol/L)	72 (61–148)	80 (63–96)	91 (63–142)	76 (61–94)
Albumin (g/L)	23 (20–27)	48 (46–50)	24 (20–29)	48 (44–50)
BCHE (ng/mL)	720 (358–1286)	3861 (3308–4553)	691 (424–949)	3966 (3198–4791
Transferrin (mg/dL)	86 (57–151)	268 (230–292)	104 (65–162)	263 (231–284)
Transthyretin (mg/dL)	51 (35–95)	215 (157–250)	60 (39–85)	222 (177–270)
Apolipoprotein AI (g/L)	0.77 (0.30–1.28)	2.6 (2.4–3.0)	0.83 (0.50–1.27)	2.8 (2.5–3.2)

Baseline characteristics are shown as frequencies or medians with interquartile ranges

### Human liver tissue

Liver tissues from nine patients who underwent liver surgery at the University of Aachen between the years 2006 and 2018 were analyzed. Unaffected surrounding portions of liver tissue from nine patients collected during oncological surgery for exclusion of liver malignancy were used as controls (Supplementary Table S1). RNA was isolated using the RNeasy tissue mini isolation kit (Qiagen, Hilden, Germany). RNA was translated to cDNA using the M-MLV reverse transcriptase kit (Promega, Madison, WI, USA) with random hexamer primers (Thermo Scientific, Waltham, MA, USA). The relative expression of genes of interest was determined using qPCR using specific primers (Supplementary Table S2). The human ribosomal gene RPLPO was used as an internal loading control.

Further experimental procedures (e.g., proteomics, bioinformatics, and statistical analysis) are given in the supplementary materials.

## Results

### Cirrhosis is associated with a profound alteration of hepatocellular serum proteins

**Fig. 1:**
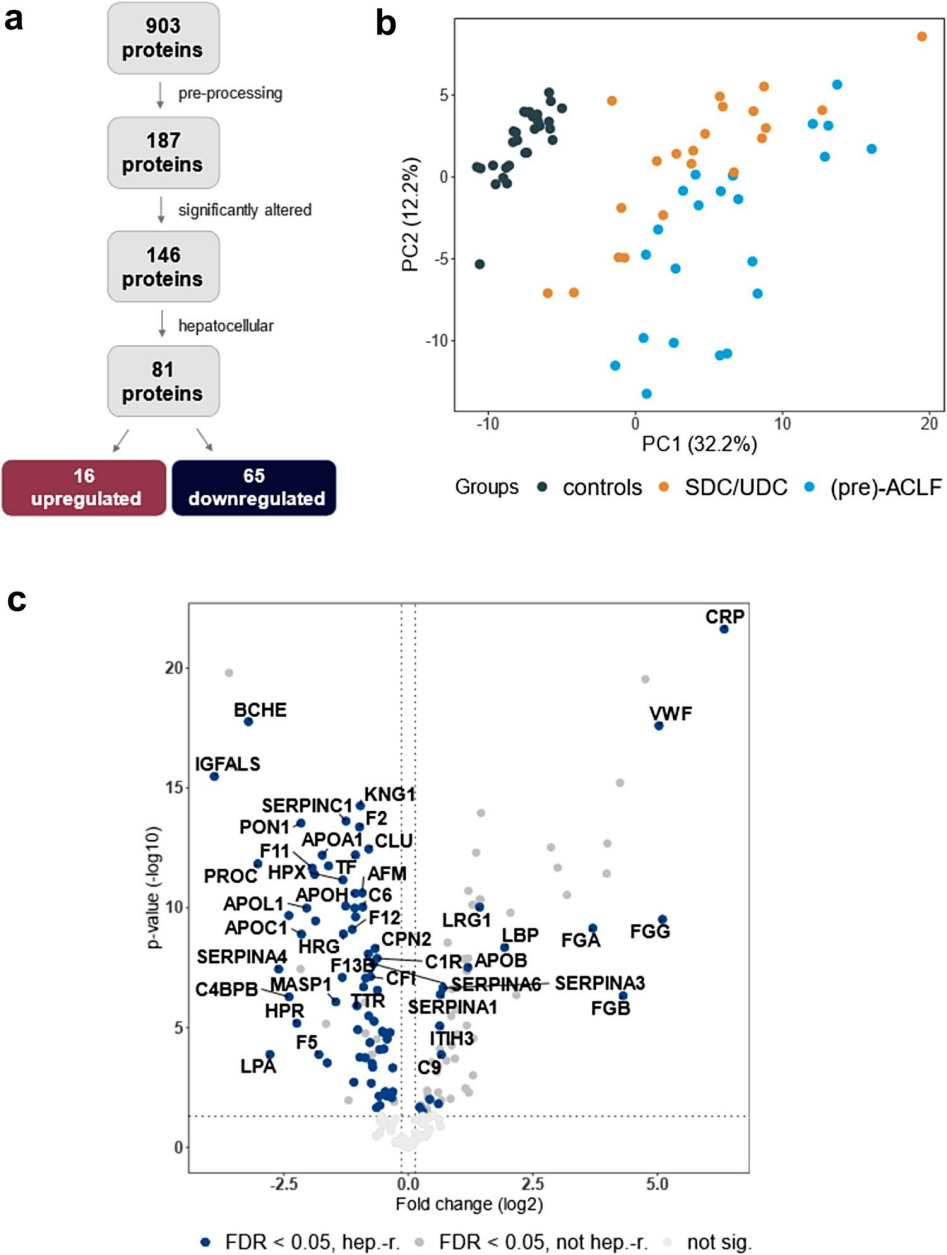
Serum proteome analysis. **a** Flow chart shows the number of detected proteins (903) and the number of proteins that were identified in a sufficient number of participants (187). One hundred and forty-six of them differentially regulated in a group of patients with decompensated cirrhosis, comprising patients with stable (SDC) and unstable decompensated cirrhosis (UDC) and (pre-)acute-on-chronic liver failure (ALCF) compared to controls (FDR < 0.05). Among those, 81 were of hepatocellular origin (16 upregulated, 65 downregulated). **b** The first two dimensions of the principal component analysis present a clear discrimination between patients with decompensated cirrhosis and healthy controls. **c** Volcano plot illustrates differentially abundant hepatocellular proteins. The − log_10_ (false discovery-adjusted *p* value) is plotted against the log_2_-fold change (resembling the differences between means of log2-transformed LFQ intensities for patients with cirrhosis vs. those of healthy controls). A log2-fold change > 0 indicates proteins upregulated in patients with cirrhosis, while a value < 0 identifies downregulated species. The non-axial vertical lines denote ± 0.05-fold change (the smallest log2-fold change observed for any significantly altered protein), while the non-axial horizontal line denotes *p* = 0.05 as the significance threshold. *hep-r* hepatocyte-related

To identify biomarkers of hepatocellular function altered in patients with cirrhosis, sera from 29 healthy individuals and 43 patients with acute decompensation of cirrhosis were subjected to untargeted proteomic analysis (Table 1). Out of 903 identified proteins, 187 were detected in at least 50% of the samples (Fig. 1A, Supplementary Table S3). Serum levels of 146 proteins differed significantly (false discovery rate (FDR) < 0.05) between groups and both groups were clearly separated by principal component analysis (Fig. 1B, Supplementary Table S4). Eighty-one of the proteins were of hepatocellular origin, and of these, sixty-five were significantly lower in sera from patients with cirrhosis as compared to healthy controls applying FDR of less than 0.05. Notably, C-reactive protein (CRP) and pseudocholinesterase (or butyrylcholinesterase; BCHE) were among the most significantly upregulated and downregulated proteins, respectively, demonstrating both loss of hepatocellular synthesis and activation of acute-phase response (Fig. 1C). On the other hand, albumin (ALB), a well-established surrogate of hepatocellular synthesis, displayed only a moderate difference between patients with cirrhosis and controls (Supplementary Table S4).

**Fig. 2:**
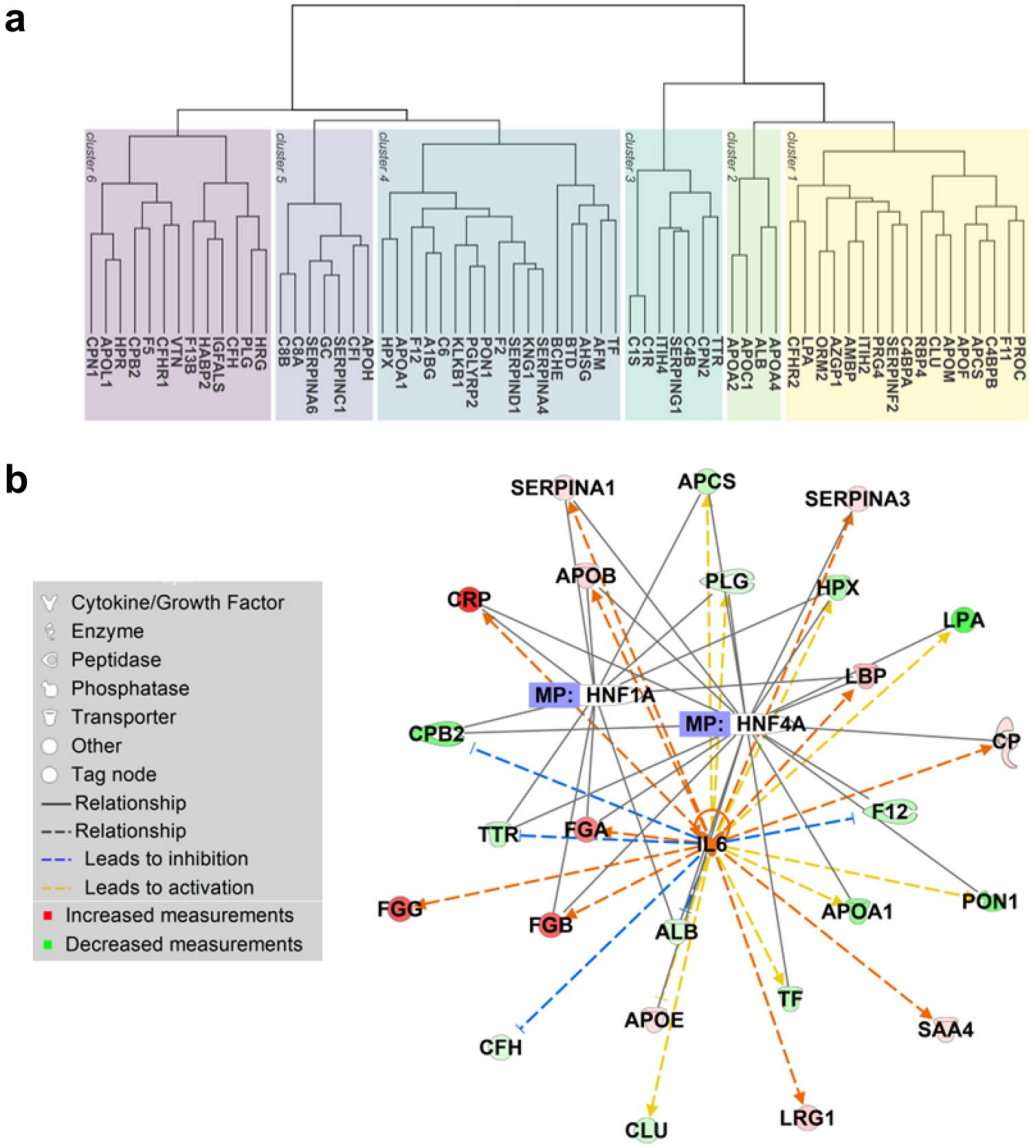
Serum proteins of hepatocellular origin decreased in decompensated cirrhosis. **a** The dendrogram indicates the hierarchical clustering of 65 serum proteins of hepatocellular origin that were significantly downregulated (log2-fold change < 0 and FDR < 0.05) in patients with cirrhosis as compared to healthy controls. Proteins are labeled via the according gene symbol. **b** Network of three most strongly enriched upstream regulators (HNF1A, HNF4A, and IL-6) and their corresponding downstream regulators as predicted by the Ingenuity Pathway Core Analysis. The analysis was based on hepatocyte-related proteins that were significantly altered (FDR < 0.05) between healthy controls and individuals with liver cirrhosis

Unsupervised hierarchical cluster analysis revealed that these 65 proteins of interest could be categorized into 2 main clusters and 6 sub-clusters (Fig. 2A, Supplementary Table S5). The number of proteins per cluster ranged from 4 to 20. Overall, the analysis demonstrated profound differences in the hepatocellular serum proteome of patients with decompensated cirrhosis when compared to controls, as shown in Supplementary Tables S5 and S6, but also the fact that hepatocellular proteins exhibit unique behavior during the development of end-stage liver disease.

### Upstream regulator analyses revealed two major upstream regulators associated with analyzed hepatocellular proteins

**Fig. 3:**
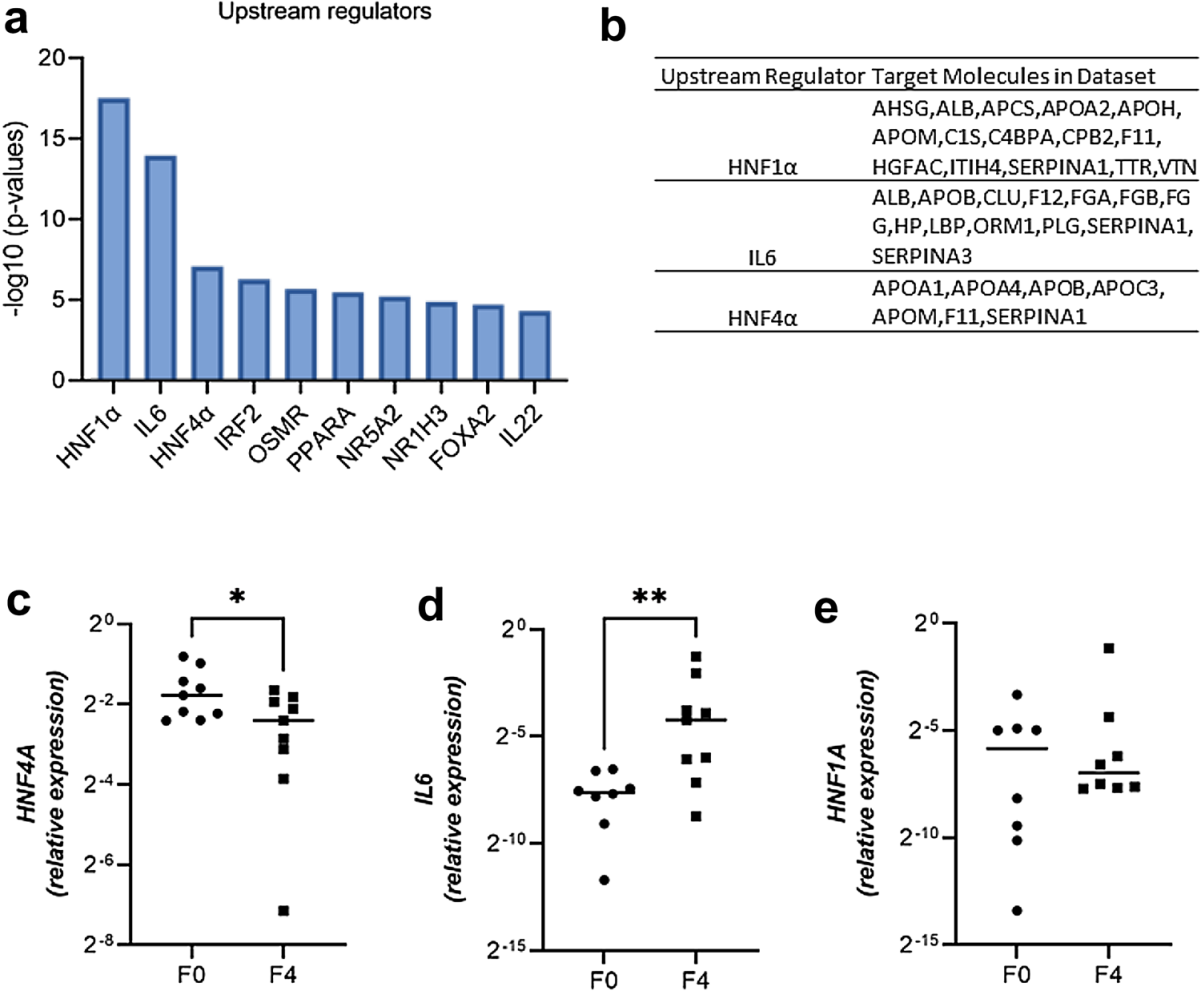
Analysis of upstream regulators altered in control vs. cirrhotic livers. **a** IPA analysis delineates the pathways altered in patients with decompensated cirrhosis vs. controls. Minus log10 (*p *values) of predicted upstream regulators are shown. **b** Target genes that are associated with top three upstream regulators obtained from IPA analysis. **C–E** Relative mRNA expression of selected genes was assessed in livers from patients with cirrhosis and non-fibrotic livers and was normalized to human ribosomal protein (RPLPO) as a housekeeping gene. Medians and individual values are shown. *p < 0.05; **p < 0.01 in non-parametric *t* test. *F0* no fibrosis, *F4* fibrosis stage 4

**Fig. 4:**
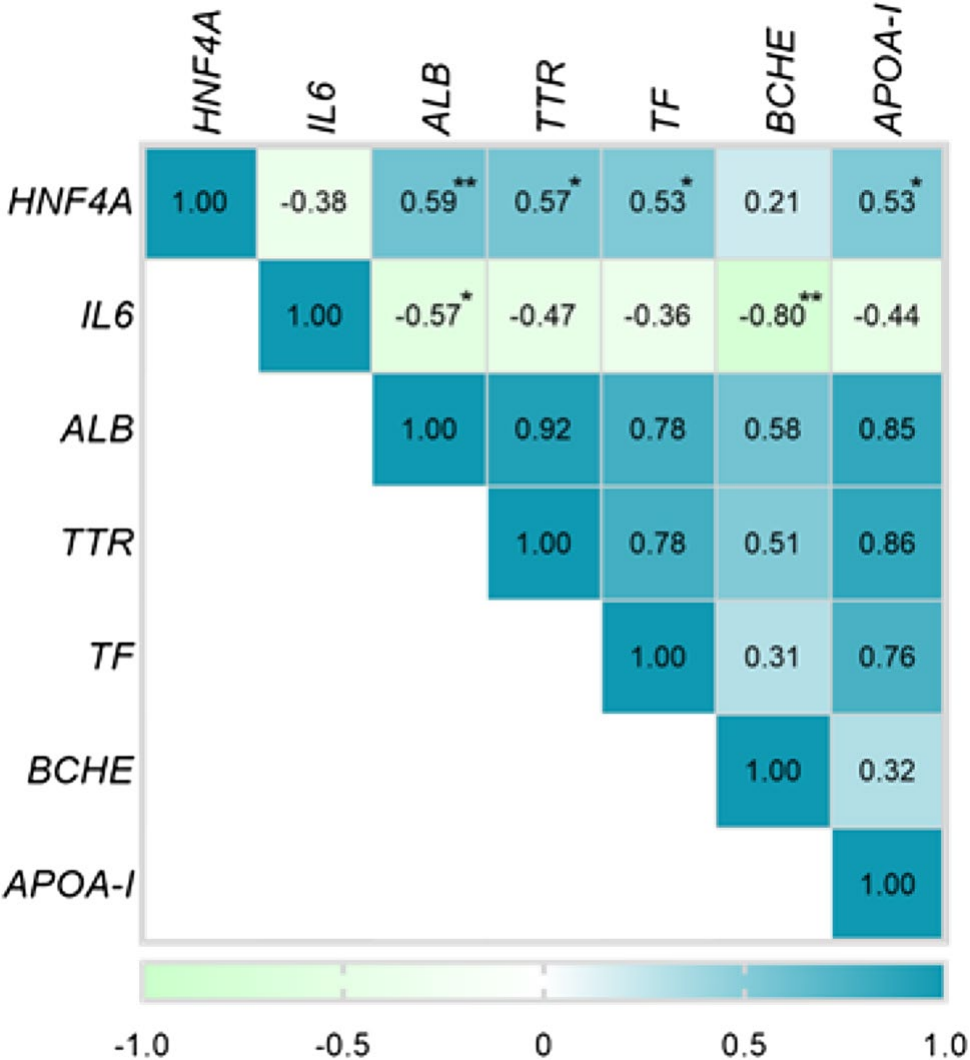
Correlation between hepatic mRNA expression of upstream regulators and their putative target genes. Spearman correlation coefficients based on RT-PCR analysis of cirrhotic and non-fibrotic livers are shown (*n* = 9 each). *p < 0.05; **p < 0.01. Proteins are labeled via the according gene symbol

To obtain an insight into the molecular mechanisms underlying serum proteome changes in decompensated cirrhosis, proteomics data were submitted to QIAGEN’s Ingenuity Pathway Analysis (IPA, QIAGEN Redwood City, www.qiagen.com/ingenuity). IPA core analysis revealed 71 upstream regulators to be significantly altered (threshold for overlap *p* value < 0.05). Hepatocyte nuclear factors 1α, 4α (HNF1α, HNF4α), and interleukin 6 (IL6) were the top three predicted transcriptional regulators that may explain the changes occurring in decompensated cirrhosis (Fig. 3A) and the predicted target proteins included albumin, apolipoproteins, transport proteins, coagulation factors, and acute-phase proteins (Fig. 3B). The suggested expression changes in *HNF4A* and *IL6* mRNA, but not *HNF1A* were validated in liver tissue of patients with cirrhosis as compared to nine controls without (Fig. 3C–E). Hepatic *HNF4A* expression positively correlated with hepatic albumin (*ALB)*, transthyretin (*TTR)*, transferrin (*TF*), and apolipoprotein AI (*Apo-AI*) expression, whereas hepatic *IL6* expression negatively correlated with hepatic *ALB* and *BCHE* expression (Fig. 4), but the correlations were not particularly strong. To further visualize the interplay between the key regulators (i.e., *HNF1A, HNF4A* and *IL6*) and the serum proteins that were altered in our proteomic dataset, we performed an IPA-driven network visualization (Fig. 2B). It demonstrated that most serum proteins are co-regulated by more of the players and that the observed changes are likely a result of a complex regulation involving several different players.

### Biomarkers of hepatocellular synthesis correlate with the trajectory of acute decompensation in cirrhosis

**Fig. 5:**
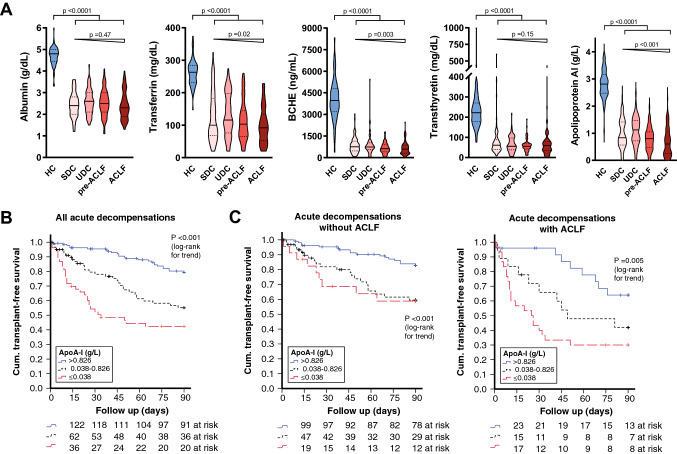
Regulated hepatocellular biomarkers and outcome in acute decompensation of cirrhosis. **a** Concentrations of albumin, transferrin, pseudocholinesterase (BCHE), transthyretin, and apolipoprotein AI in sera from healthy controls (HC) and patients with stable decompensated cirrhosis (SDC), unstable decompensated cirrhosis (UDC), pre-ACLF and ACLF depicted by violin plots. **b** Kaplan–Meier analysis of 90-days transplant-free survival stratified for apolipoprotein AI (Apo-AI). Cutoffs were derived from decision tree analysis using the Chi-square automatic interaction detectors (CHAID) algorithm with death/liver transplantation (LTX) within 90 days as events and albumin, pseudocholinesterase, transferrin, transthyretin, and apolipoprotein AI as variables. **c** Kaplan–Meier analysis of 90-days transplant-free survival stratified for Apo-AI if shown for patients with acute decompensation without (left panel) and with ACLF at baseline (right panel). *p* values from log-rank test for linear trends are indicated

Based on hierarchical cluster analysis (Supplementary Table S5) and the availability in routine clinical testing, we selected five indicator proteins, which were decreased in sera from patients with cirrhosis for further analysis. Their concentrations were analyzed in sera from 61 healthy individuals and from 285 patients with acute decompensation of cirrhosis. As expected, patients with cirrhosis had significantly lower serum concentrations of albumin, transferrin, transthyretin, pseudocholinesterase, and apolipoprotein AI compared to healthy individuals (Fig. 5A). In addition, transferrin, BCHE, and apolipoprotein AI but not albumin and transthyretin display a stage-dependent decrease of serum concentrations from stable (SDC) and unstable decompensated cirrhosis (UDC) to pre-ACLF and ACLF as indicated by a significant Jonckheere-Terpstra test (Fig. 5A).

### Higher serum levels of transthyretin are associated with better prognosis in patients with ACLF

Out of 285 patients with acute decompensation of cirrhosis (Table 1), 82 (29%) died within 90 days and 10 (4%) underwent liver transplantation. Diagnostic accuracy in predicting mortality or transplant at 90 days was compared using areas under the receiver operating characteristic curves (AUROC, supplementary table S7). Among the investigated hepatocellular biomarkers, apolipoprotein AI (AUROC 0.678; *p* = 0.0001), BCHE (AUROC 0.626; *p* = 0.001), transferrin (AUROC 0.602; *p* = 0.009), and transthyretin (AUROC 0.593; *p* = 0.01) were able to discriminate between the two groups, whereas serum albumin was not (AUROC 0.496; *p* = 0.923). The prognostic abilities of apolipoprotein AI were numerically comparable to those of the MELD score (AUROC = 0.685; *p* < 0.001). In Chi-square automatic interaction detectors (CHAID) analysis, apolipoprotein AI outperformed BCHE, transferrin, transthyretin, and albumin as predictors of death or transplant within 90 days and suggested stratification of apolipoprotein AI based on two optimized cutoffs, namely 0.380 g/L and 0.826 g/L. In Kaplan–Meier analysis, higher apolipoprotein AI serum concentrations were associated with better 90-days transplant-free survival (Fig. 5B), which remained true in the subgroups of patients with and without ACLF (Fig. 5C). In time-to-event analysis, apolipoprotein AI strata remained significant in two different multivariable Cox regression models adjusting for demographic factors and for severity of liver disease, i.e., MELD score or ACLF (Table 2).

**Table 2:** Cox regression analysis of death or transplant within 90 days

	Univariable analysis		Multivariable analysis (model 1)		Multivariable analysis (model 2)	
	Unadjusted hazard ratio (95% CI)	*p* value	Adjusted hazard ratio (95% CI)	*p* value	Adjusted hazard ratio (95% CI)	*p* value
Age	1.05 (1.03–1.07)	< 0.001	1.06 (1.04–1.09)	< 0.001	1.07 (1.04–1.09)	< 0.001
MELD score (per 1-point increase)	1.10 (1.07–1.13)	< 0.001	Not included		1.09 (1.05–1.12)	< 0.001
ACLF	2.88 (1.91–4.35)	< 0.001	2.53 (1.61–3.96)	< 0.001	Not included	
HCC at baseline	1.96 (1.18–3.24)	0.009	1.88 (1.07–3.30)	0.029	1.67 (0.96–2.91)	0.071
Apolipoprotein AI*						
> 0.826 g/L	1.00 (reference)		1.00 (reference)		1.00 (reference)	
0.381–0.826 g/L	2.67 (1.58–4.53)	< 0.001	2.72 (1.60–4.63)	< 0.001	2.27 (1.33–3.88)	0.003
≤ 0.381 g/L	4.58 (2.69–7.78)	<0.001	4.33 (2.49–7.51)	< 0.001	3.29 (1.85–5.85)	<0.001

^*^cutoff based on Chi-square automatic interaction detectors (CHAID) algorithm

## Discussion

Using untargeted serum proteomics, in silico upstream regulator analysis and validation ELISAs alongside with hepatic gene expression analysis, we herein show that serum concentrations of proteins regulated by HNF-4α-and IL-6 are reduced in cirrhosis, associated with more advanced stages of cirrhosis, and indicate poor transplant-free survival. Low serum levels of apolipoprotein AI identified patients with very low likelihood of transplant-free survival in a large cohort of patients with acute decompensation of cirrhosis. The prognostic usefulness of apolipoprotein AI seen in our study further extends and corroborates previous findings [13, 14].

The prognosis in patients with advanced chronic liver disease is mainly determined by liver function, portal hypertension, the presence of extrahepatic organ failure, and systemic inflammation [15–17]. In advanced cirrhosis, inflammation is a major driver of complications and mortality, and pro-inflammatory cytokines, acute-phase proteins, and immune activation markers have been employed to improve risk prediction in decompensated liver disease [18–21]. The identified prognostic relevant hepatocellular proteins, albumin, apolipoprotein AI, transthyretin, and transferrin, are negative acute-phase proteins, and low concentrations of negative acute-phase proteins can serve as surrogates of reduced hepatic synthesis and impaired hepatocellular reserve but also indicate the presence of systemic inflammation. Upstream regulator analysis identified HNF-4α and IL-6 as the most important regulators underlying the observed serum proteome changes in patients with decompensated cirrhosis. Whereas the hepatic mRNA expression of the principal inducer of acute-phase response, *IL6*, was increased in cirrhotic livers, hepatic mRNA expression of *HNF4A* was reduced and positively correlated with the expression of *ALB*, *TTR*, *TF* and *Apo-AI*. The promoter regions of *TTR* and other negative APP contain HNF-4α binding sites, and this correlation has been observed in patients with alcoholic hepatitis [22–24].

Although *BCHE* expression did not correlate with *HNF4A* expression, its strong negative correlation with *IL6* expression alongside the positive correlation of BCHE with transferrin and transthyretin on the protein level suggest a similar role as a negative APP and likely explains its marked decrease in patients with cirrhosis.

Despite a comparable hepatic regulation of the indicator proteins investigated in more detail, i.e., albumin, apolipoprotein AI, transthyretin, transferrin, and BCHE, their role as a prognostic biomarker somewhat differed. This may be due to different serum half-lives ranging from < 1 day (apolipoprotein AI), 2–4 days (transthyretin) across 8–12 days for transferrin and BCHE to 3 weeks (albumin). As higher levels of apolipoprotein AI identified a subgroup of patients with ACLF with better prognosis, apolipoprotein AI may be interpreted as the most dynamic marker of the hepatic reserve after liver injury and organ failure.

Hepatic HNF-4α-dependent gene expression is altered across the spectrum from fibrogenesis to decompensation and liver failure. Mechanistically, in early liver disease with fibrosis, liver matrix stiffness and cytoskeletal tension inhibit the hepatocellular HNF4-α transcriptional network [25]. In advanced liver disease, such as alcohol-related liver failure, hepatic activity of the liver-enriched transcription factor HNF-4α is severely inhibited, reducing hepatic expression of cytochrome P450 enzymes, apolipoproteins, and aldolases [26]. This process is driven by growth factors such as TGF-β, HGF, and EGF, cytokines such as TNF, IL-1β, IFN-γ, and inflammatory mediators such as PGE2 and LPS, and hepatocyte de-differentiation may play an additional role [26]. Because these upstream regulators play a prognostic role for complications of decompensated cirrhosis as well, the association of HNF-4α-regulated proteins with outcome in patients with acute decompensation of cirrhosis is plausible.

In a study on liver tissue from patients at different stages of decompensation, HNF-4α expression was downregulated and correlated with liver dysfunction, fibrosis stage, and prognostically relevant serum parameters bilirubin, albumin, and prothrombin time [27]. As a result, lower serum concentrations of transthyretin and BCHE correlated with the Child–Pugh stage and with the presence of complications such as hepatic encephalopathy in a study on patients with predominantly viral cirrhosis [28]. Plasma proteome of 459 patients with compensated alcohol-related chronic liver disease demonstrated that lower levels of plasma albumin, BCHE, and transthyretin were associated with the presence of significant liver fibrosis and hepatic inflammation (vs. no/minimal fibrosis/inflammation), but not with the degree of hepatic steatosis [29].

In this study, we demonstrate remarkable differences in serum levels of *bona fide* hepatocellular proteins as well as an association between lower apolipoprotein AI levels and transplant-free survival after adjusting for confounding factors such as age, MELD score, and the presence of hepatocellular carcinoma. Notably, the prognostic ability of apolipoprotein AI was comparable to the routinely used MELD score consisting of three different biomarkers. Moreover, apolipoprotein AI remained a significant predictor of mortality in two different multivariable models including MELD or ACLF.

These data suggest that the activity of the liver-enriched transcription factor HNF-4α as a surrogate for hepatocellular reserve, degree of fibrosis, hepatocyte differentiation, and inflammatory status may provide additional prognostic information in advanced stages of cirrhosis. However, several limitations of our work need to be considered. The observed analyses are based on a single-center cohort, and the clinical cohorts used for mRNA analyses differed from cohorts used for proteomic assessment since liver biopsies are rarely ethically justifiable in subjects with decompensated liver cirrhosis. Furthermore, the single biomarkers assessed in our study have been studied before and in this respect, our results are confirmative. The demonstrated heterogeneity in hepatocellular proteins in subjects with cirrhosis should spur large-scale proteomic studies addressing the prognostic usefulness of the individual protein patterns. Such studies might be able to identify and validate clinically useful prognostic biomarkers as it has been recently demonstrated for alcoholic liver disease [29].

## Supplementary Information

Below is the link to the electronic supplementary material.

Supplementary file1 (XLSX 204 KB)

Supplementary file2 (XLSX 34 KB)

Supplementary file3 (XLSX 33 KB)

Supplementary file4 (DOCX 50 KB)

## Data Availability

Proteomic data were deposited in a publicly available database. Further data are available from authors upon reasonable request.

## Abbreviations

APPs: Acute-phase proteins
ALB: Albumin
TF: Transferrin
TTR: Transthyretin
BCHE: Pseudocholinesterase
Apo-AI: Apolipoprotein A1
CRP: C-reactive protein
HC: Healthy controls
SDC: Stable decompensated cirrhosis
UDC: Unstable decompensated cirrhosis
ACLF: Acute-on-chronic liver failure
MELD: Model of end-stage liver disease
CHAID: Chi-square automatic interaction detectors
HNF1A and 4A: Hepatocyte nuclear factor 1 or 4 alpha
RPLPO: Human ribosomal protein
TGF-b: Transforming growth factor beta
HGF: Hepatocyte growth factor
EGF: Epidermal growth factor
TNF: Tumor necrosis factor
IL-1b: Interleukin-1 beta
IFN-g: Interferon gamma
PGE2: Prostaglandin E2
IL-6: Interleukin-6

## Declarations

### Further remarks

We attest that we did not use any copyright protected material in our manuscript. No writing assistance was provided.
