# Supplementary material for: Biomarkers of hepatocellular synthesis in patients with decompensated cirrhosis
Source: Hepatol Int. 2023 Jan 18;17(3):698–708. doi: 10.1007/s12072-022-10473-x (PMC10224844; doi:10.1007/s12072-022-10473-x)
Supplement: Supplementary file 4 — Supplementary file4 (DOCX 50 KB) [file 12072_2022_10473_MOESM4_ESM.docx]

**Supplemental material for online-only**

**Biomarkers of hepatocellular synthesis in patients with decompensated cirrhosis**

**Berivan Gurbuz^1*^, Nurdan Guldiken^1,*^, Philipp Reuken^2^, Lei Fu^1,3^, Katharina Remih^1^, Christian Preisinger^4^, Radan Brůha^5^, Martin Leníček^6^, Jaromír Petrtýl^5^, Johanna Reissing^1^, Mahmoud Aly^1,7^, Malin Fromme^1^, Biaohuan Zhou^1, 8^, Isabel Karkossa^9^, Kristin Schubert^9^ , Martin von Bergen^9,10,11^ , Andreas Stallmach^2^, Tony Bruns^1,2**^, Pavel Strnad^1,**^**

**^1^**Medical Department III, Gastroenterology, Metabolic diseases and Intensive Care, University Hospital RWTH Aachen, Aachen, Germany; **^2^**Department of Internal Medicine IV, Jena University Hospital, Friedrich Schiller University, Jena, Germany; **^3^**Department of Science and Technology, Ruikang Hospital Affiliated to Guangxi University of Chinese Medicine, Nanning 530011, Guangxi Zhuang Autonomous Region, China; **^4^** Proteomics Facility, Interdisciplinary Center for Clinical Research (IZKF), University Hospital RWTH Aachen, Germany; **^5^**4th Department of Internal Medicine, First Faculty of Medicine and General University Hospital in Prague, Charles University, Prague, Czech Republic; **^6^**Institute of Medical Biochemistry and Laboratory Diagnostics, First Faculty of Medicine and General University Hospital in Prague, Charles University, Prague, Czech Republic; **^7^**Department of Medicine and Infectious Diseases, Faculty of Veterinary Medicine, University of Sadat 12 City, Sadat City, Egypt; **^8^**Department of Surgical Oncology, Fujian Provincial Hospital, Fuzhou, China; **^9^**Department of Molecular Systems Biology, Helmholtz Centre for Environmental Research, Leipzig, Germany; **^10^**German Centre for Integrative Biodiversity Research,(iDiv) Halle-Jena-Leipzig, Leipzig, Germany; **^11^**University of Leipzig, Faculty of Life Sciences, Institute of Biochemistry, Leipzig, Germany

**The authors share first* and last** authorship.**

**Corresponding author:**

Pavel Strnad, MD

Department of Internal Medicine III and IZKF, University Hospital Aachen

Address: Pauwelsstraße 30, D-52074 Aachen, Germany

Phone: +49 (241) 80-35324, Fax: +49 (241) 80-82455

E-mail: pstrnad@ukaachen.de

**Supplementary experimental procedures**

**Serum proteomics**

For proteome analysis, sera were separated on SDS-PAGE gradient gels (4-20%; Serva, Heidelberg, Germany) and stained with Coomassie Brilliant Blue: 5 gel slices were excised from each lane and the proteins were digested in-gel using trypsin as described previously S(1). The resulting peptides were then desalted using homemade C18 tips and dried in a speed vac. Prior to mass-spectrometry analysis, the samples were resuspended in 15 μl 3% formic acid (FA)/5% acetonitril (ACN) and then loaded onto an LC-system (RSLCnano, Thermo Scientific). Mass spectrometry analysis was in principle carried out as described in a previous study S(2). The peptides were first trapped on a C18 precolumn (Acclaim PepMap100, C18, 5 μm, 100 Å, 300 μm i.d. × 5 mm, Thermo Scientific, Bremen, Germany) using buffer A (0.1% FA) and then separated on a C18 analytical column (Easyspray (ES902): 2 μm particle size, 75 μm inner diameter, 25 cm length, 45°C column oven temperature, 2 kV; Thermo Scientific) coupled with the Easyspray (Thermo Scientific) source using a 70 minutes gradient. The settings were: 0–10 minutes, 5% buffer B (80% ACN/0.1% FA); 10–42 minutes, 5%–35% B; 42–47 minutes, 35%–99% B; 47-52 minutes, 99% B; 52–55 minutes, 99%–5% B; 55–70 minutes, 5% B. The mass spectrometry analysis was done on a Q Exactive Plus instrument (Thermo Scientific) using the data-dependent mode. Full Q Exactive settings: resolution: 70000; AGC target: 3e6; maximum injection time: 100 milliseconds; scan range, 300–1650 m/z. dd-MS2 settings: resolution: 17500 ; AGC target: 2e5; maximum injection time: 110 milliseconds; precursor fragmentation: top 10; isolation window: 1.8 m/z; collision energy: 27. dd settings: minimum AGC: 5e2; dynamic exclusion: 10s; only 2+ to 5+ peptides allowed.

The raw data was analysed using MaxQuant (MQ version 2.0.1.0; S(3)) using the built-in Andromeda search engine and a human Uniprot database (April 2021, only canonical and reviewed sequences). In general, MQ default settings were employed. Specific settings: Trypsin as specific protease (with 2 missed cleavages allowed); fixed modification: carbamidomethylation; variable modifications: oxidation (on methionine residues) and N-terminal protein acetylation; false-discovery-rate: 0.01 on both protein and peptide level; minimum peptide length: 7 amino acids. The MQ label-free (LFQ) algorithm was used for quantification. LFQ-quantification was done without normalization.

The resulting proteinGroups.txt file from MaxQuant was analyzed and filtered using Perseus (version 1.6.14.0; S(4)). First, all “reverse” and “only identified by site” entries were removed. Potential contaminants were removed manually. Proteins were required to be identified with a minimum of 1 unique and 2 (razor + unique) peptides to be considered for further analysis.

**Data availability**

The mass spectrometry proteomics data have been deposited to the ProteomeXchange Consortium via the PRIDE S(5) partner repository with the dataset identifier PXD035024. Reviewer account details: Username: [reviewer_pxd035024@ebi.ac.uk](mailto:reviewer_pxd035024@ebi.ac.uk); Password: nAnwGBCB

**Bioinformatic analysis of proteomics**

Both Perseus program (4), and *R* (R Foundation, Vienna, Austria) were then used for statistical analysis of the mass spectrometry data. Data were log2-transformed and proteins which could be quantified in at least 50 % of all samples were selected. To identify outliers, group-wise coefficients of variation were calculated for each identified protein and features lower than 0.3 in at least one of the subgroups (healthy controls or patients with cirrhosis) were considered for further downstream analysis. Missing data were imputed column-wise with random draws from a distribution of the leftmost tail of the data that was based on a quantile regression of the data (QRILC algorithm) S(6) and further the values were mean-centred. A Student t-test t-test was performed to compare between different samples. P values were false discovery rate (FDR)-adjusted employing the Benjamini-Hochberg method, and P value less than 0.05 were considered significant.

Unsupervised hierarchical clustering was conducted by applying pair-wise distance measures between all included variables and was based upon their similarity provided by a Euclidean distance matrix. Linkage of clusters was determined via the Ward method S(7). Analyses were conducted using the software packages imputeLCMD S(8), “dpylr” S(9), “factoextra” S(10), “ggplot2” S(11), “ggpubr” S(12), “cluster” S(13), “NbClust” S(14),“viridis” S(15), “Hmisc” S(16), and “ggcorrplot” S(17). Correlations between selected biomarkers were tested in pairwise manner using spearman’s rank correlation test. The Benjamini-Hochberg method was used to adjust for multiple testing. Functional annotation of regulated proteins, as well as prediction of enriched pathways and their corresponding upstream regulators were performed using Ingenuity Pathway Analysis (IPA, Qiagen), resulting in p-values from Fisher’s Exact Test reflecting the significance of upstream regulator enrichment.

**Serum protein quantitation**

Measurement of serum albumin (ALB) and transferrin (TF) (turbidimetry) using Cobas 8000 system (Roche Diagnostics, Mannheim, Germany) was performed by the Clinical Chemistry Department of University Hospital Aachen. Concentrations of serum, human transthyretin (TTR, Prealbumin; Avivasysbio, OKIA00081, San Diego, CA, USA), human pseudocholinesterase (BCHE, Butyrylcholinesterase; R&D systems DBCHE0, Minneapolis, MN, USA) and human apolipoprotein A1 (APOA1; R&D systems DAPA10, Minneapolis, MN, USA) were measured using commercial sandwich ELISAs.

**Statistical analysis**

Statistical analyses were performed using SPSS v25 and v28 (IBM) and visualized using GraphPad Prism 9 (GraphPad, La Jolla, CA, USA). Wilcoxon–Mann–Whitney U test or Student’s t-tests were used to compare differences between continuous variables from two groups. Jonckheere-Terpstra test was used to compare concentrations between groups with increasing severity of decompensation. The diagnostic ability of biomarkers to predict death or transplantation within 90 days was tested by receiver operating characteristics (ROC) analysis. Dichotomization of variables was performed by decision tree analysis using the chi-square automatic interaction detectors (CHAID) algorithm with death or transplant mortality as independent variable. Transplant-free survival was displayed with Kaplan-Meier curves and groups were compared using log-rank tests, and univariable and multivariable Cox regression analyses were performed. Patients were right-censored at loss to follow-up or after 90 days. All tests were evaluated using two-sided testing and p values were considered significant when p<0.05.

**Supplementary table S1. Characteristics of control subjects and individuals with advanced fibrosis used for gene expression analyses.**

|  | **Controls**  **(n=9)** | **Cirrhosis**  **(n=9)*** |
| --- | --- | --- |
| Gender (M/F) | 3/6 | 6/3 |
| Age | 58 (38-61) | 54 (46-60) |
| Etiology | tumor-free liver from patients  with malignant liver disease | 5 HCV; 2 ALD; 1 NASH; 1 other |

* This cohort includes patients who underwent liver transplantation. Baseline characteristics are shown as frequencies or medians with interquartile ranges.

Abbreviations: ALD, alcoholic liver disease; HCV, chronic hepatitis C infection; NASH, Non-alcoholic steatohepatitis.

**Supplementary table S2. Primer sequences for gene expression analysis**

| **Gene** | **Primer sequence** |
| --- | --- |
| Human hepatocyte nuclear factor 4 alpha *(HNF4A)* | F: ACTCTCCAAAACCCTCGTCG  R: TCGAGGCACCGTAGTGTTTG |
| Human hepatocyte nuclear factor 1 alpha *(HNF1A)* | F: TGAGCAAAGAGGCACTGATCC  R: GCGTGAAGTCTTCCCCATCG |
| Human interleukin 6 (*IL6)* | F: TAGTGAGGAACAAGCCAGAGC  R: TGGGTCAGGGGTGGTTATTG |
| Human ribosomal protein (*RPLPO*) | F: GCAATGTTGCCAGTGTCTGT  R: GCCTTGACCTTTTCAGCAAG |

**Supplementary Table S3.** File contains the raw MaxQuant output which was used for all further downstream analysis, as well as a table with the pre-processed LFQ intensities following the steps described in the *Patients and* *Methods* section. ***[see excel file]***

**Supplementary Table S4.** List of all 187 proteins identified in the proteomic analysis. The table comprises the total number of identifications for each protein, group-wise means, log2-fold changes, and (adjusted) p-values stemming from a Welch t-test to compare study groups. P-values were adjusted employing the Benjamini-Hochberg method. ***[see excel file]***

**Supplementary Table S5.** List of significantly regulated biomarkers of hepatocellular synthesis obtained in the hierarchical clustering (Figure 2). The analysis included 29 healthy controls and 43 patients with cirrhosis. Proteins were labelled via the according gene symbol. *Representative biomarkers that were selected for further analysis.

| **cluster 1** | **cluster 2** | **cluster 3** | **cluster 4** | **cluster 5** | **cluster 6** |
| --- | --- | --- | --- | --- | --- |
| *PROC* | *APOA4* | ***TTR**** | ***TF**** | *APOH* | *HRG* |
| *F11* | *APOA2* | *C1S* | *HPX* | *C8B* | *CPN1* |
| *C4BPB* | *APOC1* | *C1R* | ***APOA1**** | *C8A* | *APOL1* |
| *APCS* | ***ALB**** | *ITIH4* | *F12* | *SERPINA6* | *HPR* |
| *APOF* |  | *SERPING1* | *A1BG* | *GC* | *CPB2* |
| *APOM* |  | *C4B* | *C6* | *SERPINC1* | *F5* |
| *CLU* |  | *CPN2* | *KLKB1* | *CFI* | *CFHR1* |
| *RBP4* |  |  | *PGLYRP2* |  | *VTN* |
| *C4BPA* |  |  | *PON1* |  | *F13B* |
| *SERPINF2* |  |  | *F2* |  | *HABP2* |
| *PRG4* |  |  | *SERPIND1* |  |  |
| *ITIH2* |  |  | *KNG1* |  |  |
| *AMBP* |  |  | *SERPINA4* |  |  |
| *AZGP1* |  |  | ***BCHE**** |  |  |
| *ORM2* |  |  | *BTD* |  |  |
| *LPA* |  |  | *AHSG* |  |  |
| *CFHR2* |  |  | *AFM* |  |  |

**Supplementary Table S6.** List of 81 significantly regulated biomarkers of hepatocellular synthesis by untargeted proteomic analysis. The table comprises the total number of identifications for each protein, group-wise means, log2-fold changes, and (adjusted) p-values stemming from a Welch t-test to compare study groups. P-values were adjusted employing the Benjamini-Hochberg method**. *[see excel file]***

**Supplementary Table S7.** Diagnostic accuracy in predicting mortality. Areas under the receiver operating characteristic curves (AUROC) and their 95% confidence intervals (CIs) are shown.

|  | AUROC (95% CIs; lower limit-upper limit) | P values |
| --- | --- | --- |
| Apolipoprotein AI (g/L) | 0.678 (0.606-0.749) | <0.001 |
| Albumin (g/L) | 0.496 (0.419-0.573) | 0.923 |
| BCHE (ng/mL) | 0.626 (0.551-0.701) | 0.001 |
| Transferrin (mg/dL) | 0.602 (0.525-0.678) | 0.009 |
| Transthyretin (mg/dL) | 0.593 (0.520-0.666) | 0.012 |

**Supplementary references**

S1. von Kriegsheim A, Preisinger C, Kolch W. Mapping of signaling pathways by functional interaction proteomics. Methods Mol Biol 2008;484:177-192.

S2. Dusterhoft S, Kahveci-Turkoz S, Wozniak J, Seifert A, Kasparek P, Ohm H, Liu S, et al. The iRhom homology domain is indispensable for ADAM17-mediated TNFalpha and EGF receptor ligand release. Cell Mol Life Sci 2021;78:5015-5040.

S3. Tyanova S, Temu T, Cox J. The MaxQuant computational platform for mass spectrometry-based shotgun proteomics. Nat Protoc 2016;11:2301-2319.

S4. Tyanova S, Temu T, Sinitcyn P, Carlson A, Hein MY, Geiger T, Mann M, et al. The Perseus computational platform for comprehensive analysis of (prote)omics data. Nat Methods 2016;13:731-740.

S5. Perez-Riverol Y, Bai J, Bandla C, Garcia-Seisdedos D, Hewapathirana S, Kamatchinathan S, Kundu DJ, et al. The PRIDE database resources in 2022: a hub for mass spectrometry-based proteomics evidences. Nucleic Acids Res 2022;50:D543-D552.

S6. Wei R, Wang J, Su M, Jia E, Chen S, Chen T, Ni Y. Missing Value Imputation Approach for Mass Spectrometry-based Metabolomics Data. Sci Rep 2018;8:663.

S7. Ward JHJ. Hierarchical Grouping to Optimize an Objective Function. Journal of the American Statistical Association 1963:236-244.

S8. Lazar C. imputeLCMD: A collection of methods for left-censored missing data imputation. R package version 2.0. 2015.

S9. Wickham H, François R, Henry H, Müller K. dplyr: A Grammar of Data Manipulation. R package version 1.0.9. . 2022.

S10. Kassambara A, Mundt F. factoextra: Extract and Visualize the Results of Multivariate Data Analyses. R package version 1.0.7. . 2020.

S11. Wickham H. ggplot2: Elegant Graphics for Data Analysis. . 2016.

S12. Kassambara A. ggpubr: 'ggplot2' Based Publication Ready Plots. R package version 0.4.0.

S13. Maechler M, Rousseeuw P, Struyf A, Hubert M, Hornik K. cluster: Cluster Analysis Basics and Extensions. R package version 2.1.2. 2021.

S14. Charrad M, Ghazzali N, Boiteau V, Niknafs A, . NbClust: An R Package for Determining the Relevant Number of Clusters in a Data Set. . Journal of Statistical Software:1-36.

S15. Garnier S, Ross N, Rudis R, Camargo AP, Sciaini M, Scherer C. Rvision -Colorblind-Friendly Color Maps for R. R package version 0.6.2. 2021.

S16. Harrell FEJ. Hmisc: Harrell Miscellaneous. R package version 4.7-0. . 2022.

S17. Kassambara A. ggcorrplot: Visualization of a Correlation Matrix using 'ggplot2'. R package version 0.1.3. 2019.
